# *Reldesemtiv* in Patients with Spinal Muscular Atrophy: a Phase 2 Hypothesis-Generating Study

**DOI:** 10.1007/s13311-020-01004-3

**Published:** 2021-02-23

**Authors:** Stacy A. Rudnicki, Jinsy A. Andrews, Tina Duong, Bettina M. Cockroft, Fady I. Malik, Lisa Meng, Jenny Wei, Andrew A. Wolff, Angela Genge, Nicholas E. Johnson, Carolina Tesi-Rocha, Anne M. Connolly, Basil T. Darras, Kevin Felice, Richard S. Finkel, Perry B. Shieh, Jean K. Mah, Jeffrey Statland, Craig Campbell, Ali A. Habib, Nancy L. Kuntz, Maryam Oskoui, John W. Day

**Affiliations:** 1 Cytokinetics, Incorporated, South San Francisco, CA USA; 2 Columbia University, New York, NY USA; 3 Stanford University, Stanford, CA USA; 4 Sangamo Therapeutics, Brisbane, CA USA; 5 Montreal Neurological Institute, Montreal, QC Canada; 6 Virginia Commonwealth University, Richmond, VA USA; 7 University of Utah, Salt Lake City, UT USA; 8 Nationwide Children’s Hospital, Columbus, OH USA; 9 Washington University, St Louis, MO USA; 10 Boston Children’s Hospital and Harvard Medical School, Boston, MA USA; 11 Hospital for Special Care, New Britain, CT USA; 12 Nemours Children’s Hospital, Orlando, FL USA; 13 St. Jude Children’s Research Hospital, Memphis, TN USA; 14 University of California, Los Angeles, Los Angeles, CA USA; 15 University of Calgary, Alberta Children’s Hospital, Calgary, AB Canada; 16 University of Kansas, Lawrence, KS USA; 17 Department of Pediatrics, Epidemiology and Clinical Neurological Sciences, University of Western Ontario, London Health Sciences Centre, London, ON Canada; 18 University of California, Irvine, Orange, CA USA; 19 Ann & Robert H. Lurie Children’s Hospital of Chicago, Chicago, IL USA; 20 McGill University Health Centre Research Institute, Montreal, QC Canada

**Keywords:** *Reldesemtiv*, spinal muscular atrophy clinical trial, pharmacodynamics, pharmacokinetics, six-minute walk test

## Abstract

This phase 2, double-blind, placebo-controlled, hypothesis-generating study evaluated the effects of oral *reldesemtiv*, a fast skeletal muscle troponin activator, in patients with spinal muscular atrophy (SMA). Patients ≥ 12 years of age with type II, III, or IV SMA were randomized into 2 sequential, ascending *reldesemtiv* dosing cohorts (cohort 1: 150 mg bid or placebo [2:1]; cohort 2: 450 mg bid or placebo [2:1]). The primary objective was to determine potential pharmacodynamic effects of *reldesemtiv* on 8 outcome measures in SMA, including 6-minute walk distance (6MWD) and maximum expiratory pressure (MEP). Changes from baseline to weeks 4 and 8 were determined. Pharmacokinetics and safety were also evaluated. Patients were randomized to *reldesemtiv* 150 mg, 450 mg, or placebo (24, 20, and 26, respectively). The change from baseline in 6MWD was greater for *reldesemtiv* 450 mg than for placebo at weeks 4 and 8 (least squares [LS] mean difference, 35.6 m [*p* = 0.0037] and 24.9 m [*p* = 0.058], respectively). Changes from baseline in MEP at week 8 on *reldesemtiv* 150 and 450 mg were significantly greater than those on placebo (LS mean differences, 11.7 [*p* = 0.038] and 13.2 cm H_2_O [*p* = 0.03], respectively). For 6MWD and MEP, significant changes from placebo were seen in the highest *reldesemtiv* peak plasma concentration quartile (*C*_max_ > 3.29 μg/mL; LS mean differences, 43.3 m [*p* = 0.010] and 28.8 cm H_2_O [*p* = 0.0002], respectively). Both dose levels of *reldesemtiv* were well tolerated. Results suggest *reldesemtiv* may offer clinical benefit and support evaluation in larger SMA patient populations.

**Supplementary Information:**

The online version contains supplementary material available at 10.1007/s13311-020-01004-3.

## Introduction

Spinal muscular atrophy (SMA) is a genetic neuromuscular disease in which reduced amounts of functional survival motor neuron (SMN) protein result in progressive muscle weakness and atrophy, leading to various degrees of functional motor impairment [1]. Analyses of markers of muscle involvement, including contractile and regulatory proteins, suggest a delay in muscle maturation in samples from patients with SMA compared to controls [2, 3]. Magnetic resonance imaging of muscle in children and adults aged 2 to 45 years with SMA type II and III showed differences with varying degrees of proximal to distal gradient, but found some muscle groups were always spared and muscles involved demonstrated atrophy in both SMA types [4].

Although SMA has historically been classified based on age of onset and highest level of motor function achieved [1], in the advent of recently approved therapies, this classification system has become less relevant. All three recently approved SMA course-modifying treatments increase motor neuron SMN protein content. Nusinersen, an antisense oligonucleotide drug that increases SMN production by altering *SMN2* splicing [5, 6], was the first approved medication for the specific treatment of SMA. The beneficial effects of nusinersen in infants and children are well substantiated [5–8], but currently there are limited data available from older patients; findings for nusinersen in adults are limited to observational studies rather than controlled clinical trials [9, 10]. The gene therapy onasemnogene abeparvovec-xioi is effective in infants and recently gained approval by the US Food and Drug Administration for patients < 2 years of age with SMA [11, 12]. In addition, risdiplam, a small molecule *SMN2* splicing modifier, is also now approved to treat patients 2 months and older with SMA, and has been studied in a randomized, double-blind, placebo-controlled study in patients up to the age of 25 [13, 14]. Even after treatment with these new therapies, many very young SMA patients fail to meet age-specific motor milestones, and treatment later in the course of disease results in even less dramatic benefits.

Therefore, there continue to be unmet medical needs despite these dramatic advances, particularly regarding persistent weakness, impaired endurance, and fatigue [8, 12]. As new emerging treatments for SMA impact the development of motor milestones, and as treatment before manifestation of symptoms may be possible through newborn screening, the clinical features of SMA are likely to slowly evolve. Other features such as *SMN2* copy number, and age of onset of signs and symptoms may also impact clinical course [15, 16], but even as the clinical landscape of SMA evolves, the vast majority of patients will continue to experience weakness and fatigue.

*Reldesemtiv* (CK-2127107) is being developed to improve skeletal muscle function for disease states involving muscle weakness or fatigue; it selectively binds to the fast skeletal troponin complex and sensitizes it to calcium [17]. In a double-blind, randomized, placebo-controlled, phase 1 clinical study in healthy participants, fibular nerve stimulation at sub-tetanic frequencies resulted in significantly increased placebo-corrected changes in the force generated by the tibialis anterior muscle in a dose-, concentration-, and frequency-dependent manner following *reldesemtiv* treatment. In this and 2 additional double-blind, randomized, placebo-controlled, phase 1 studies, *reldesemtiv* was well tolerated at single-dose levels from 30 to 4000 mg and multiple doses of 300 and 500 mg twice daily (bid) [18]. Adverse events (AEs) observed in ≥ 10% of patients in at least one of the studies included headache, dizziness, nausea, and asthenopia (eye strain); all AEs were mild or moderate in severity. No meaningful differences in pharmacokinetic (PK) parameters were found between young and elderly volunteers.

We conducted a hypothesis-generating, phase 2 study with no pre-specified primary outcome measure that tested a number of pharmacodynamic (PD) outcomes. Two oral dose levels (150 and 450 mg bid) of *reldesemtiv* were evaluated in patients with SMA. Secondary objectives were to evaluate the safety and tolerability of *reldesemtiv* as well as its PK properties.

## Methods

### Study Design

The study was a phase 2, double-blind, randomized, placebo-controlled, multiple-dose investigation of *reldesemtiv* in 2 sequential ascending dose cohorts of patients with SMA (ClinicalTrials.gov identifier NCT02644668), conducted from December 2015 through May 2018 at 18 centers in Canada and the USA. The study received approval from institutional review boards before commencing, and was conducted in compliance with good clinical practice and the Declaration of Helsinki; written informed consent was obtained from patients ≥ 18 years of age, and parental permission and child assent were obtained for those < 18 years of age. Patients were randomized via an interactive web response system to receive *reldesemtiv* 150 mg bid or placebo (2:1; cohort 1), or *reldesemtiv* 450 mg bid or placebo (2:1; cohort 2), stratified by ambulatory status. The study drug was constituted with water at the site; the suspension was given to the patient for oral administration (9 mL) bid, approximately every 12 h after a ≥ 3-h fast, with a 1-h fast following dosing, for a total of 8 weeks.

### Study Participants

Patients eligible for enrollment had genetically confirmed diagnosis of type II, III, or IV SMA and were ≥ 12 years of age. Ambulatory patients, after independently achieving a standing position, were required to complete at least 1 lap (≥ 50 m) in the 6-minute walk test (6MWT) without assistance. Non-ambulatory patients needed to be able to tolerate an upright supported sitting position continuously for 3 h, and required a wheelchair for mobility needs, though they may have been able to stand or walk less than 50 m without assistance in 6 min. Patients were also required to have forced vital capacity (FVC) > 20% predicted, Hammersmith Functional Motor Scale Expanded (HFMSE) score ≥ 10 and ≤ 54, and contracture of elbow flexion ≤ 90 degrees. Patients must have been able to swallow an oral suspension and were expected to be able to continue to do so for the duration of the study. Prior or concomitant treatment with nusinersen was not allowed.

### Outcome Measures

Outcome measures were assessed at screening, day 1, at the end of weeks 1, 2, 4, and 8, and at follow-up (4 weeks after the last dose) for all except timed up and go (TUG) and 6MWT, which were assessed at screening, day 1, at the end of weeks 4 and 8, and at follow-up. The Spinal Muscular Atrophy-Health Index (SMA-HI) was completed on day 1 and at the end of week 8 in cohort 2 only. All clinical measures were standardized with certified clinical evaluators who underwent appropriate training to ensure consistency of measures across all sites and visits throughout the study.

Pulmonary function was assessed via FVC, as well as maximum inspiratory pressure (MIP) and maximum expiratory pressure (MEP), measured using calibrated spirometers by Micro Direct, Inc., Lewiston, ME (FVC: MicroLab Spirometer MK8; MIP and MEP: MicroRPM). Isometric muscle strength was assessed bilaterally using a make test, in which the examiner holds the dynamometer in a set position as the patient pushes against it, for 3 muscle groups (elbow flexion, knee flexion, and shoulder abduction) with a MicroFET2 handheld dynamometer (HHD, Hoggan Scientific, LLC, Salt Lake City, UT). The maximum muscle strength of 2 measurements was reported as percent change from baseline, with imputed muscle strength set to missing if the baseline value was 0. The mega-score, a composite score for strength across the 3 muscle groups, was calculated as the mean of transformed muscle strength scores. The HFMSE was used to evaluate functional mobility with a scale score ranging from 0 to 66, with higher scores reflecting better function [19]. The revised upper limb module (RULM) was designed to be used in conjunction with the HFMSE; it tests upper limb function based on reachable workspaces from upper (shoulder), middle (elbow), and distal (wrist and hand) regions of the upper limb with a range of scoring from 0 to 44, with higher scores reflecting greater function [20].

The 6MWT, performed in ambulatory patients, measured the distance a patient walked in 6 min (6MWD) in order to assess functional endurance capacity and mobility; fatigue was measured by the difference in distance walked between the first and last minutes [21]. To assess mobility, balance, and walking ability, the TUG, performed in ambulatory patients, measured the time for a patient to rise from a chair, traverse 3 m, turn around, return to the chair, and sit down [22]. Patient-reported SMA burden was measured using the SMA-HI questionnaire, with higher scores reflecting greater burden of disease; this was performed only in cohort 2 given the timing of when the questionnaire was fully developed [23]. Safety was assessed by monitoring AEs, coded using the Medical Dictionary for Regulatory Activities (MedDRA), version 18.0, clinical laboratory findings, and electrocardiogram intervals.

For PK endpoints, blood samples for determination of plasma concentrations of *reldesemtiv* were collected prior to dosing on day 1 and at the ends of weeks 1, 2, 4, and 8. Concentrations were determined by a validated solid-phase extraction method using high-performance liquid chromatography followed by tandem mass spectrometric detection.

### Statistical Analysis

The safety population consisted of all patients who received ≥ 1 dose of study drug, whereas the PD population included patients in the safety population with ≥ 1 post-baseline endpoint assessment, and the PK population included those in the safety population with ≥ 1 evaluable PK level. Baseline characteristics and safety data were summarized descriptively overall and by treatment, dose level, and ambulatory status in the safety population.

For dose-response PD effects, the change from baseline in a continuous endpoint was analyzed by repeated measures mixed effect models that accounted for within-patient correlation with an unstructured covariance matrix or compound symmetry when an unstructured covariance matrix could not converge. The covariates included dose level, visit, interaction between dose level and visit, ambulatory status, and the baseline value of the variable being analyzed (except for muscle strength mega-score, for which the score itself represents a percent change from baseline). Ambulatory status was removed from the model for subgroup analyses by ambulatory status. Important baseline characteristics (including age, gender, and age of disease onset) were also examined and included in the model.

The slope of change from baseline for continuous endpoints was analyzed by a mixed effect model with no intercept, and included dose level, days from the first dose of study treatment as a random covariate, interaction of dose level-by-days from the first dose of study treatment, ambulatory status, and baseline value of the variable being analyzed.

Maximum observed plasma concentration (*C*_max_), predose plasma concentration (*C*_trough_), and area under the plasma concentration-time curve from 0 to 12 h (AUC_0–12_) were calculated based on the plasma concentrations of *reldesemtiv* using noncompartmental PK methods and were summarized descriptively. For concentration-response PK analyses, the change in continuous endpoints from baseline was analyzed by a mixed effect model that accounted for within-patient correlation with an unstructured covariance matrix. The covariates included PK parameter, visit, interaction between visit and the PK parameter, ambulatory status, and the baseline value of the variable being analyzed.

For model-based PD analyses, least squares (LS) mean, difference of LS means between *reldesemtiv* and placebo (combined from the 2 cohorts), their standard errors (SEs) and 95% confidence intervals (CIs), and 2-sided *p* values were determined. Multiplicity was not addressed in this hypothesis-generating study; all *p* values of statistical significance are nominal.

## Results

### Patient Disposition and Baseline Characteristics

Seventy patients were randomized to placebo (*n* = 26), *reldesemtiv* 150 mg bid (*n* = 24), or *reldesemtiv* 450 mg bid (*n* = 20) (Supplemental Fig. 1). Of the 44 patients randomized to *reldesemtiv*, 41 (93.2%) completed the 8-week study. Three patients (6.8%), all in the 450 mg group, discontinued treatment early due to an AE, a protocol violation, or withdrawal by the patient; the latter 2 also withdrew from the study. Twenty-four of the 26 patients (92.3%) in the placebo group completed the study; 2 patients (7.7%) discontinued treatment early due to an AE but completed the planned visits for those who terminated from treatment early. One additional patient completed dosing but was lost to follow-up and did not complete the final follow-up study visit.

Baseline demographics and PD measures were generally similar across treatment groups (Table 1). The overall mean age was 29.4 years (range 12–72 years), and the majority of patients were male (58.6%) and white (90.0%). Most (91.4%) patients had type III SMA (none had type IV); 44.3% were ambulatory.

**Table 1:** Baseline demographics, disease characteristics, and pharmacodynamic measures

	Placebo (*n* = 26)	*Reldesemtiv* 150 mg bid (*n* = 24)	*Reldesemtiv* 450 mg bid (*n* = 20)	Overall (*n* = 70)
Age, years, mean (SD)	28.5 (16.0)	27.8 (12.0)	32.6 (17.9)	29.4 (15.3)
Age < 18 years, *n* (%)	8 (30.8)	7 (29.2)	5 (25.0)	20 (28.6)
Male, *n* (%)	15 (57.7)	14 (58.3)	12 (60.0)	41 (58.6)
White, *n* (%)	22 (84.6)	23 (95.8)	18 (90.0)	63 (90.0)
BMI, mean (SD)	24.3 (7.4)	25.4 (9.2)	25.1 (5.5)	24.9 (7.5)
SMA type II, *n* (%)	2 (7.7)	3 (12.5)	1 (5.0)	6 (8.6)
SMA type III, *n* (%)	24 (92.3)	21 (87.5)	19 (95.0)	64 (91.4)
Age at SMA onset, years, mean (SD)	3.8 (4.6)	7.8 (6.9)	8.1 (9.4)	6.4 (7.2)
Ambulatory, *n* (%)	11 (42.3)	12 (50.0)	8 (40.0)	31 (44.3)
% predicted FVC, mean (SD)	84.4 (22.4)	83.1 (22.0)	85.9 (21.2)	84.4 (21.7)
MEP, cm H_2_O, mean (SD)	86.5 (36.9)	94.0 (43.4)	88.9 (47.7)	89.8 (41.8)
MIP, cm H_2_O, mean (SD)	− 105.7 (38.5)	− 109.1 (44.2)	− 101.0 (43.2)	− 105.6 (41.3)
HFMSE score, mean (SD)	30.6 (16.6)	36.0 (17.2)	30.4 (16.3)	32.4 (16.7)
RULM total score, mean (SD)	31.0 (8.7)	34.8 (7.9)	33.7 (8.0)	33.1 (8.3)
TUG, s, mean (SD)	21.5 (11.0)	15.7 (6.5)	22.8 (16.1)	19.2 (10.3)
6MWD, m, mean (SD)	240.1 (111.8)	316.6 (69.0)	311.0 (107.3)	287.3 (99.2)
SMA-HI total score, mean (SD)^a^	33.1 (19.9)	NA	39.8 (17.1)	37.5 (18.1)

^a^SMA-HI was only performed in participants in cohort 2

*6MWD* 6-minute walking distance, *BMI* body mass index, *bid* twice daily, *FVC* forced vital capacity, *HFMS-E* Hammersmith Functional Motor Score-Expanded, *MEP* maximum expiratory pressure, *MIP* maximum inspiratory pressure, *NA* not assessed, *RULM* revised upper limb module, *SD* standard deviation, *SMA-HI* Spinal Muscular Atrophy-Health Index, *TUG* timed up and go

### Outcomes

**Fig. 1:**
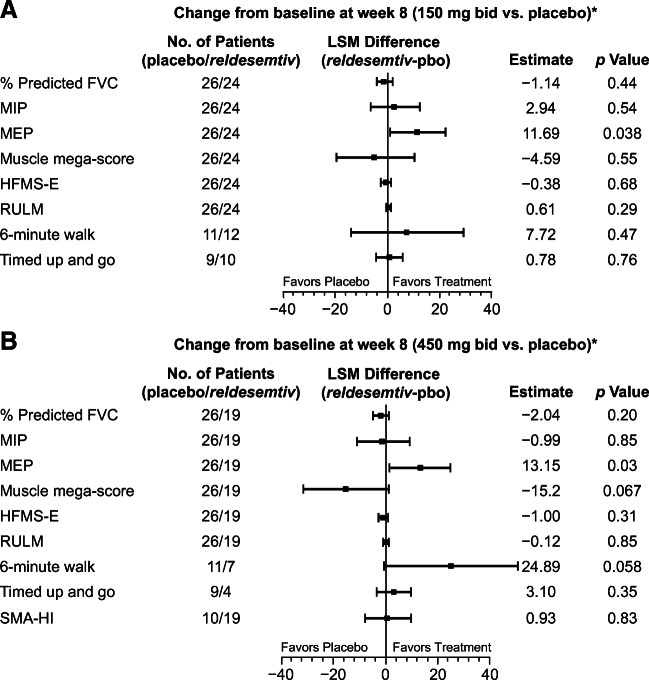
Least squares mean change in outcome measures from baseline to week 8 in participants receiving *reldesemtiv* at doses of (A) 150 mg bid, and (B) 450 mg bid compared with placebo. *LSM change on *reldesemtiv* minus LSM change on placebo for all measures except TUG and SMA-HI, which are LSM change on placebo minus LSM change on *reldesemtiv*. bid = twice daily; FVC = forced vital capacity; HFMS-E = Hammersmith Functional Motor Score-Expanded; LSM = least squares mean; MEP = maximum expiratory pressure; MIP = maximum inspiratory pressure; pbo = placebo; RULM = revised upper limb module; SMA-HI = Spinal Muscular Atrophy-Health Index; TUG = timed up and go

Changes from baseline to week 8 for most of the PD parameters were not significantly different from placebo in either *reldesemtiv* dose group with the exception of the 6MWT and MEP (Fig. 1).

**Fig. 2:**
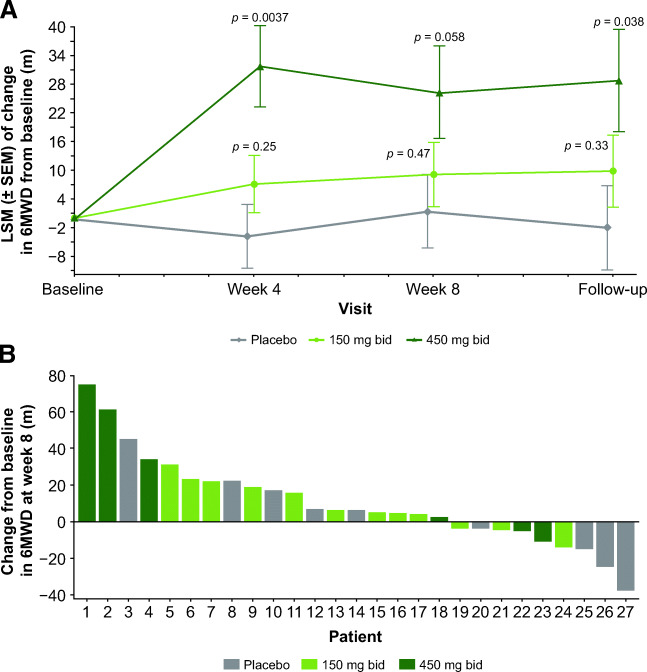
(A) Changes from baseline in 6MWD over time and (B) waterfall plot of individual changes from baseline to week 8 in 6MWD. 6MWD = 6-minute walking distance; bid = twice daily; LSM = least squares mean; m = meters; SEM = standard error of the mean

The 6MWD increased in both the *reldesemtiv* 150-mg and 450-mg dose groups at weeks 4 and 8 and was fairly stable in the placebo arm (Fig. 2(A)). The LS mean difference was significantly greater than placebo at week 4 for the *reldesemtiv* 450 mg bid group (35.63 m, *p* = 0.0037); at week 8, the difference was 24.89 m (*p* = 0.058). A nominally significant LS mean difference of 30.8 m (*p* = 0.038) between *reldesemtiv* 450 mg bid and placebo was observed up to 4 weeks after dosing at the follow-up visit. Older age appeared to be related to larger increases in 6MWD from baseline to week 8 (*p* = 0.058). A waterfall plot of individual changes from baseline to week 8 demonstrates that 13 of the 18 *reldesemtiv*-treated patients and 5 of the 9 placebo-treated patients had some improvement in 6MWD (Fig. 2(B)); of the 5 patients with the greatest improvement, 4 were on *reldesemtiv*.

**Fig. 3:**
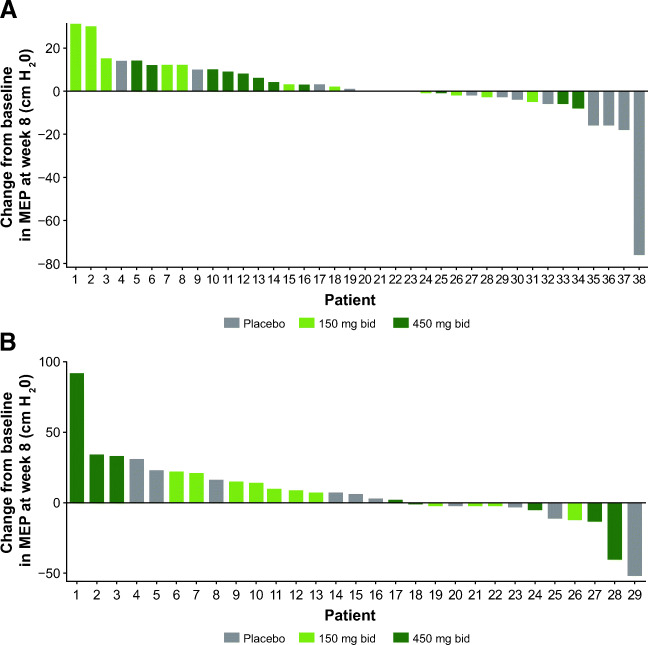
Waterfall plots of individual changes in MEP from baseline to week 8 in (A) ambulatory and (B) non-ambulatory patients. MEP = maximum expiratory pressure; bid = twice daily

MEP showed significant LS mean differences versus placebo for *reldesemtiv* 150 mg bid (11.69 cm H_2_O, *p* = 0.038, Fig. 1(A)) and for *reldesemtiv* 450 mg bid (13.15 cm H_2_O, *p* = 0.03, Fig. 1(B)). A lower baseline MEP appeared to be related to a larger increase in MEP from baseline to week 8 (*p* = 0.062). Waterfall plots of individual changes from baseline to week 8 in MEP by ambulatory status show that responses to *reldesemtiv* were observed in both ambulatory and non-ambulatory patients (Fig. 3(A, B)). In addition, baseline values in the non-ambulatory patients were lower, whereas the ambulatory patients with higher values may have been limited by a ceiling effect.

Responses to the SMA-HI questionnaire to measure patient-reported burden at 8 weeks showed no difference between the *reldesemtiv* 450 mg and placebo groups (*p* = 0.83, Fig. 1(B)).

### Pharmacokinetics

**Fig. 4:**
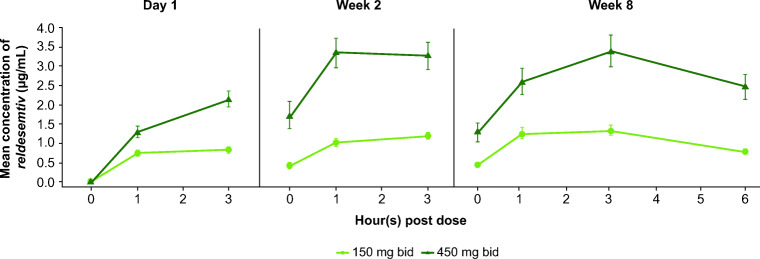
Plasma concentrations of *reldesemtiv* over time. Error bars represent standard errors. bid = twice daily

Plasma concentrations of *reldesemtiv* increased by dose (Fig. 4). Within each dose group, plasma concentrations at weeks 2 and 8 were similar. Assessment of PK parameters at week 8 showed dose-dependent increases in *C*_max_ and drug exposure (Table 2).

**Table 2:** Summary of pharmacokinetic parameters for each dose of *reldesemtiv* at week 8

Parameter, geometric mean (geometric % CV)	*Reldesemtiv* 150 mg bid (*n* = 23)	*Reldesemtiv* 450 mg bid (*n* = 15)^a^
*C*_max_, μg/mL	1.40 (43.18)	3.34 (48.77)
*C*_trough_, μg/mL	0.34 (88.27)	0.94 (98.06)
AUC_0–12_, h × μg/mL	9.00 (49.38)	25 (52.19)

^a^For *C*_trough_, *n* = 17

*AUC*_*0–12*_ area under the plasma concentration curve from 0 to 12 h, *bid* twice daily, *C*_*max*_ maximum observed plasma concentration, *C*_*trough*_ predose plasma concentration, *CV* coefficient of variation

#### Pharmacodynamic Response in Relation to Pharmacokinetics

**Fig. 5:**
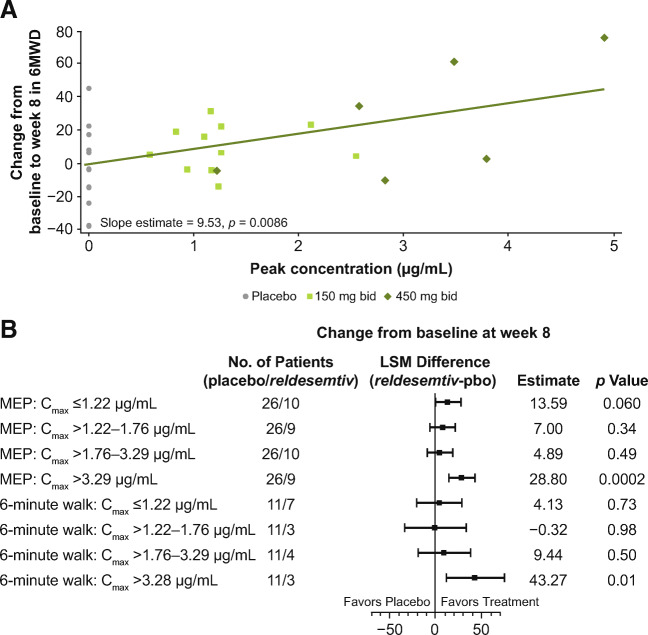
(A) Individual changes from baseline to week 8 in 6MWD by *C*_max_, and (B) changes from baseline to week 8 in MEP and 6MWD by quartiles of *C*_max_. *Difference between placebo and *reldesemtiv*. 6MWD = 6-minute walking distance; C_max_ = maximum observed plasma concentration; LSM = least squares mean; MEP = maximum expiratory pressure; pbo = placebo

Individual changes from baseline to week 8 in 6MWD by *C*_max_ were examined (Fig. 5(A)). The slope of the line shows that the 6MWD increased as *C*_max_ increased (*p* = 0.0086). When examined by quartiles of *C*_max_, statistically significant and clinically meaningful changes from baseline to week 8 were observed in the highest quartile of *C*_max_ (> 3.29 μg/mL) for MEP (*p* = 0.0002) and 6MWD (*p* = 0.010) (Fig. 5(B)).

### Safety

AEs were reported in 92.3%, 83.3%, and 85.0% of patients in the placebo, *reldesemtiv* 150 mg bid, and *reldesemtiv* 450 mg bid groups, respectively (Table 3). Most AEs were mild or moderate in severity. The most frequently reported AEs in the placebo and combined *reldesemtiv* groups were headache (19.2% and 25.0%), nausea (19.2% and 11.4%), and fatigue (15.4% and 9.1%, respectively).

**Table 3:** Adverse events reported in ≥ 2 patients treated with *reldesemtiv*

Preferred term^a^, *n* (%)	Placebo (*n* = 26)	*Reldesemtiv* 150 mg bid (*n* = 24)	*Reldesemtiv* 450 mg bid (*n* = 20)	All *reldesemtiv* (*n* = 44)
Patients with AEs	24 (92.3)	20 (83.3)	17 (85.0)	37 (84.1)
Headache	5 (19.2)	6 (25.0)	5 (25.0)	11 (25.0)
Nausea	5 (19.2)	3 (12.5)	2 (10.0)	5 (11.4)
Fatigue	4 (15.4)	2 (8.3)	2 (10.0)	4 (9.1)
Fall	3 (11.5)	1 (4.2)	1 (5.0)	2 (4.5)
Nasopharyngitis	3 (11.5)	3 (12.5)	0	3 (6.8)
Upper respiratory tract infection	4 (15.4)	0	2 (10.0)	2 (4.5)
Constipation	0	3 (12.5)	2 (10.0)	5 (11.4)
Diarrhea	2 (7.7)	2 (8.3)	1 (5.0)	3 (6.8)
Abdominal pain upper	1 (3.8)	2 (8.3)	0	2 (4.5)
Dyspepsia	0	2 (8.3)	1 (5.0)	3 (6.8)
Contusion	0	2 (8.3)	0	2 (4.5)
Respiratory tract congestion	0	2 (8.3)	0	2 (4.5)
Serum creatine kinase increased	0	0	2 (10.0)	2 (4.5)
Skin abrasion	0	0	2 (10.0)	2 (4.5)
Decreased appetite	1 (3.8)	1 (4.2)	1 (5.0)	2 (4.5)
Hypoesthesia	0	1 (4.2)	1 (5.0)	2 (4.5)
Respiratory tract infection	0	1 (4.2)	1 (5.0)	2 (4.5)
Patients with AEs resulting in early treatment termination	2 (7.7)	0	1 (5.0)	1 (2.3)
Serum creatine kinase increased	0	0	1 (5.0)	1 (2.3)
Asthenia	1 (3.9)	0	0	0
Gait disturbance	1 (3.9)	0	0	0
Muscular weakness	1 (3.9)	0	0	0

^a^Medical Dictionary for Regulatory Activities (version 18.0)

*AE* adverse event

Investigator-assessed serious AEs (SAEs) were reported for 3 patients: 2 in the *reldesemtiv* 150 mg bid group (gastroenteritis *Escherichia coli*, gastroenteritis *Salmonella*, and myocarditis in 1 patient and facial pain and hypesthesia for the other) and 1 in the *reldesemtiv* 450 mg bid group (traumatic fracture). The sponsor also upgraded the AEs of another patient in the *reldesemtiv* 450 mg bid group to SAEs (serum aspartate aminotransferase and creatine kinase increased). All SAEs resolved and none were considered to be related to the study drug. Two patients in the placebo group and 1 in the *reldesemtiv* 450 mg bid group reported AEs that led to early treatment termination (Table 3); all were considered by the treating investigator to be related to the study drug. No deaths were reported during the study.

## Discussion

In this hypothesis-generating study, 6MWD and MEP improved versus placebo in patients with SMA receiving *reldesemtiv*, consistent with increased skeletal muscle force production by *reldesemtiv*. The LS mean change from baseline in the 6MWD at week 4 was significantly greater for *reldesemtiv* 450 mg bid compared with placebo, and difference at week 8 trended to conventional statistical significance. There was a persistent effect in the 6MWD 4 weeks after the last dose; a similar persistent benefit in slow vital capacity was also observed in the FORTITUDE-ALS phase 2 trial of *reldesemtiv* in amyotrophic lateral sclerosis (ALS) patients [24], and in a phase 2 ALS trial with *tirasemtiv*, a first-generation fast skeletal muscle activator [25]. MEP measurements, which reflect expiratory muscle strength, demonstrated a nominal increase at week 8 for both *reldesemtiv* treatment groups compared with placebo. Changes seen in MEP but not in FVC may be related to the well-known relative preservation of the diaphragm compared to other ventilatory muscles in SMA [26]. Significant changes from placebo at week 8 were seen in 6MWD and MEP in the highest quartiles of *C*_max_ for *reldesemtiv*. These results are consistent with those from phase 1 studies of *reldesemtiv* in healthy volunteers in which significant dose- and frequency-dependent increases were observed in the peak force of tibialis anterior muscle contraction after deep fibular nerve stimulation following treatment with *reldesemtiv* [18].

As adult SMA patients have historically not been included in most interventional SMA trials, this trial offers insights into the challenges encountered with the commonly used SMA outcome measures in this older patient population. Because *reldesemtiv* is known to increase skeletal muscle force in response to neural input, outcome measures specific to fatigue and muscle force may be more sensitive to changes due to treatment. The HFMSE was developed for children with SMA, and includes crawling and arising from the floor, which are infrequently performed by adults, resulting in their assessment being impacted by a motor learning effect in addition to weakness from SMA. The HFMSE also includes no timed tests, so is insensitive to changes in stamina or fatigue. More sensitive outcome measures are needed to address these domains that have been reported as important to the adult SMA patient population. Potential alternatives to the HFMSE include the Revised Hammersmith (RHS) and Motor Function Measurement 32 (MFM-32) assessments. The RHS includes 2 timed tests, though it still includes crawling and kneeling. Testing has been done in the RHS for a small number of patients over the age of 14 [27]. The MFM-32 was developed for use in patients aged 6 to 60 years and does not require the patient to kneel or crawl. It has the additional benefit of having the items classified into 3 domains: D1 for standing and transfers, D2 for axial and proximal motor function and D3 for distal motor function. In an ambulatory patient population, D1 items are likely to be most impacted, whereas D2 may or may not be abnormal depending on the degree of their limb and trunk weakness. This permits response to an intervention to be measured both for the overall MFM-32 score as well as the scores of the individual domains [28–30].

The SMA-HI questionnaire was used to assess patient-reported disease burden as an exploratory outcome. No difference was observed between *reldesemtiv* and placebo treatment groups at week 8. The SMA-HI was added to the protocol for the second cohort only. Because some sites were delayed in getting approval for the revised protocol and consent form, only 19 patients filled out the SMA-HI, limiting the ability to draw meaningful conclusions from this initial investigation, though a patient-reported outcome measure will clearly be of value for treatment of unmet needs in this patient population.

PK exposures in this study were below levels that were well tolerated and associated with increased PD activity compared to placebo in the phase 1 studies (that used a different drug formulation). The *reldesemtiv* dose levels tested here, 150 mg and 450 mg administered twice daily, were also well tolerated and associated with increased PD activity compared with placebo treatment. The AEs reported were generally similar to those in the placebo group and were consistent with those observed in phase 1 studies of *reldesemtiv* [18]. The good tolerability of these dose levels, as well as no evidence of an efficacy plateau in this trial, and the prior safety and tolerability of higher exposures that generated even larger PD effects in the PD study provide a rationale to study higher dose levels in an effort to increase exposures.

The small population size and relatively short treatment period are limitations of this study. In addition, the results for MEP at 8 weeks were nominally statistically significant for both doses of *reldesemtiv* compared to placebo; for the 6MWD, only the higher dose of *reldesemtiv* was nominally statistically significant compared to placebo at 4 weeks and trended towards significance at 8 weeks. In a post hoc analysis of baseline features, a trend to predicting a response in MEP was limited to a lower baseline MEP value and only older age showed a trend to predicting a response in the 6MWD.

However, the purpose of this trial was to generate hypotheses to test in future studies and so it had no predetermined clinical primary endpoint. Good tolerability and the observation that 6MWD and MEP increased with plasma concentrations support future studies of *reldesemtiv* in larger populations of individuals with SMA. As *reldesemtiv* has a distinctly different mechanism of action compared to currently available therapies for SMA that increase levels of the SMN protein, in a future *reldesemtiv* trial, we anticipate including eligibility criteria permitting current or past use of such medicines with enrolled patients stratified accordingly. In conclusion, we believe the promising effects of *reldesemtiv* on 6MWD and MEP, in association with tolerability of the doses employed in this trial, support further clinical development of *reldesemtiv* for the potential treatment of patients with SMA.

## Supplementary Information

ESM 1 (PDF 1.19 mb)

ESM 2 (PDF 338 kb)

ESM 3 (PDF 121 kb)
