# Supplementary material for: Reldesemtiv in Patients with Spinal Muscular Atrophy: a Phase 2 Hypothesis-Generating Study
Source: Neurotherapeutics. 2021 Feb 23;18(2):1127–36. doi: 10.1007/s13311-020-01004-3 (PMC8423982; doi:10.1007/s13311-020-01004-3)
Supplement: Supplementary file 2 — (PDF 338 kb) [file 13311_2020_1004_MOESM2_ESM.pdf]

SUPPLEMENTAL MATERIALS

Reldesemtiv in Patients with Spinal Muscular Atrophy: A Phase 2 Hypothesis-Generating Study

Stacy A. Rudnicki[*] • Jinsy A. Andrews[†a] • Tina Duong[‡] • Bettina M. Cockroft[§a] • Fady I. Malik[*] • Lisa Meng[*] • Jenny Wei[*] • Andrew A. Wolff[*] • Angela Genge[¶] • Nicholas E. Johnson[||b] • Carolina Tesi-Rocha[‡] • Anne M. Connolly[#c] • Basil T. Darras[**] • Kevin Felice[††] • Perry B. Shieh[‡‡] • Jean K. Mah[§§] • Jeffrey Statland[¶¶] • Craig Campbell[||||] • Ali A. Habib[##] • Nancy L. Kuntz[***] • Maryam Oskoui[†††] • John W. Day[‡]

[*]Cytokinetics, Inc., South San Francisco, CA, USA
[†]Columbia University, New York, NY, NY
[‡]Stanford University, Stanford, CA, USA
[§]Sangamo Therapeutics, Brisbane, CA, USA
[¶]Montreal Neurological Institute, Montreal, QC, Canada
[||]Virginia Commonwealth University, Richmond, VA, USA
[#]Nationwide Children's Hospital, Columbus, OH, USA
[**]Boston Children's Hospital, Boston, Harvard Medical School, MA, USA
[††]Hospital for Special Care, New Britain, CT, USA
[‡‡]University of California, Los Angeles, Los Angeles, CA, USA
[§§]University of Calgary, Alberta Children's Hospital, Calgary, AB, USA
[¶¶]University of Kansas, Lawrence, KS, USA
[||||]Department of Pediatrics, Epidemiology and Clinical Neurological Sciences, University of Western Ontario, London Health Sciences Centre, London, ON, USA
[##]University of California, Irvine, Orange, CA, USA
[***]Ann & Robert H. Lurie Children's Hospital of Chicago, Chicago, IL, USA
[†††]McGill University Health Centre Research Institute, Montreal, QC, USA.

Affiliation during the conduct of the study: [a]Cytokinetics, Inc., South San Francisco, CA, USA; [b]University of Utah, Salt Lake City, UT, USA; [c]Washington University, St Louis, MO, USA.

Supplemental Fig. 1 Patient disposition.

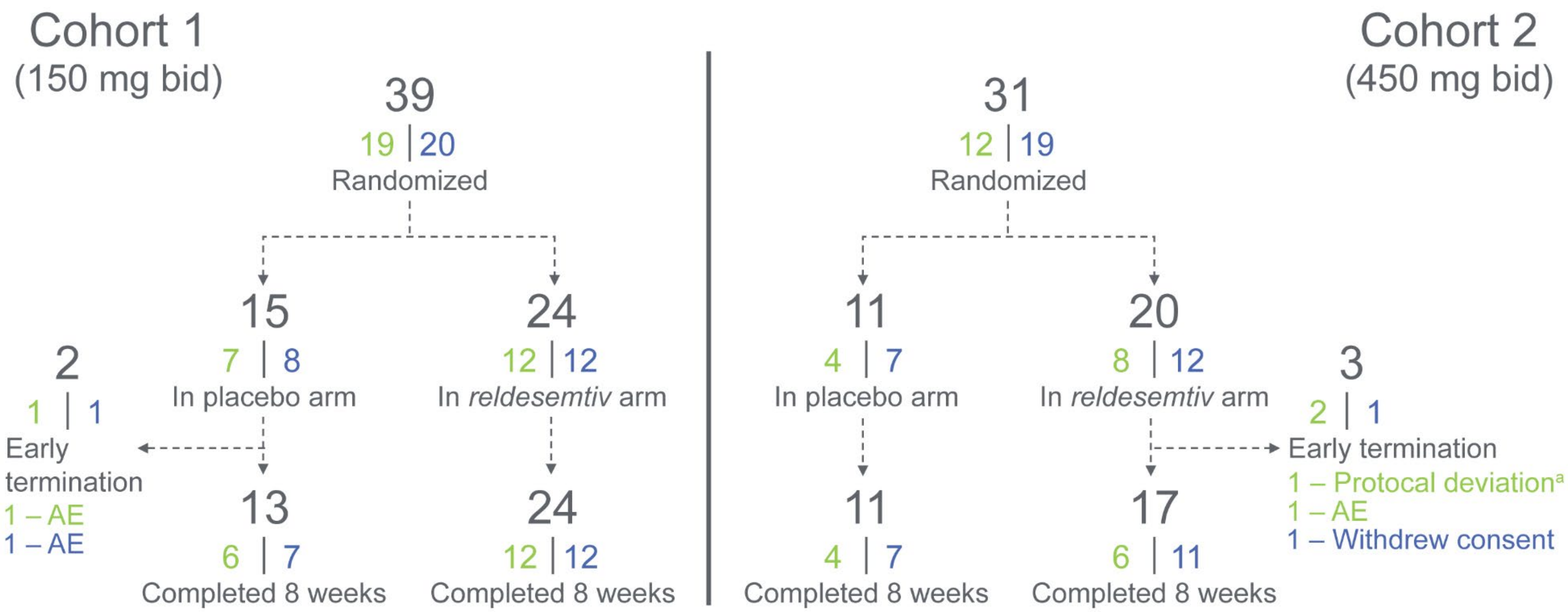

[a] Took prohibited medication.

AE = adverse event; bid, twice daily; black text = total population; green text = ambulatory patients; blue text = nonambulatory patients.
