# Supplementary material for: Reldesemtiv in Patients with Spinal Muscular Atrophy: a Phase 2 Hypothesis-Generating Study
Source: Neurotherapeutics. 2021 Feb 23;18(2):1127–36. doi: 10.1007/s13311-020-01004-3 (PMC8423982; doi:10.1007/s13311-020-01004-3)
Supplement: Supplementary file 3 — (PDF 121 kb) [file 13311_2020_1004_MOESM3_ESM.pdf]

**CY 5021 Study Group: Principal Investigators**

| Location* | Name | Email |
|---|---|---|
| **Canada** | | |
| Alberta Children's Hospital, Calgary, AB | Jean K. Mah | jean.mah@albertahealthservices.ca |
| BC Children's Hospital, Vancouver, BC | Kathryn Selby | kselby@cw.bc.ca |
| Children's Hospital - London Health Sciences Centre, London, ON | Craig Campbell | craig.campbell@lhsc.on.ca |
| Montreal Neurological Institute, Montreal, QC | Angela Genge | angela.genge@mcgill.ca |
| McGill University Health Centre Research Institute, Montreal, QC | Maryam Oskoui | maryam.oskoui@mcgill.ca |
| **United States** | | |
| University of California, Irvine, Irvine, CA | Ali A. Habib | aahabib@uci.edu |
| University of California, Los Angeles, Los Angeles, CA | Perry B. Shieh | pshieh@mednet.ucla.edu |
| Stanford University, Stanford, CA | John W. Day | jwday@stanford.edu |
| Stanford University, Stanford, CA | Carolina Tesi-Rocha | ctesiroc@stanford.edu |
| University of Colorado Denver, Aurora, CO | Stacy Dixon | stacy.dixon@ucdenver.edu |
| Children's Hospital Colorado, Aurora, CO | Julie Parsons | julie.parsons@childrenscolorado.org |
| Hospital for Special Care, New Britain, CT | Kevin Felice | kfelice@hfsc.org |
| Nemours Children's Hospital, Orlando, FL | Richard Finkel | Richard.Finkel@nemours.org |
| Ann & Robert H. Lurie Children's Hospital of Chicago, Chicago, IL | Nancy L. Kuntz | nkuntz@luriechildrens.org |
| University of Kansas, Lawrence, KS | Jeffrey Statland | JStatland@kumc.edu |
| Boston Children's Hospital, Boston, MA | Basil T. Darras | Basil.Darras@childrens.harvard.edu |
| Johns Hopkins Medicine, Baltimore, MD | Thomas Crawford | tcrawfo@jhmi.edu |
| Washington University in Saint Louis, St. Louis, MO | Anne M. Connolly | anne.connolly@nationwidechildrens.org |

| | | |
|---|---|---|
| Duke University, Durham, NC | Edward Smith | edward.smith@duke.edu |
| Ohio State University, Columbus, OH | John Kissel | John.Kissel@osumc.edu |
| Children's Hospital of Philadelphia, Philadelphia, PA | John Brandsema | brandsemaj@email.chop.edu |
| University of Utah, Salt Lake City, UT | Nicholas E. Johnson | nicholas.johnson@vcuhealth.org |

* Location at the time of conduct of the study
